# Does denosumab exert a protective effect against COVID-19? Results of a large cohort study

**DOI:** 10.3389/fendo.2023.1283101

**Published:** 2023-12-07

**Authors:** Sara Cassibba, Silvia Ippolito, Silvia Pellegrini, Roberto Trevisan, Alessandro Rossini

**Affiliations:** ^1^ Endocrinology and Diabetes Unit, Azienda Socio Sanitaria Territoriale Papa Giovanni XXIII, Bergamo, Italy; ^2^ Department of Medicine and Surgery, University of Milano Bicocca, Milan, Italy

**Keywords:** denosumab, COVID-19, SARS-CoV-2, RANKL, osteoporosis, osteoimmunology

## Abstract

**Introduction:**

Denosumab is a monoclonal antibody blocking the receptor activator of nuclear factor kappa-B/receptor activator of nuclear factor kappa-B ligand (RANK/RANKL) pathway, thus inhibiting osteoclastogenesis. Since RANK and RANKL are also involved in the immune system activation, denosumab might interfere with the response against infections. Our study aimed to explore the relationship between denosumab treatment and coronavirus disease 2019 (COVID-19).

**Design and methods:**

The occurrence and severity of COVID-19 were recorded in consecutive patients referred to the Endocrinology Department of Papa Giovanni XXIII Hospital, Bergamo, from 1 January 2020 to 1 January 2021. Patients treated with denosumab were compared to outpatient controls. Patients’ features were summarized by descriptive statistics. Multivariate logistic regression assessed the relationship between denosumab and COVID-19, adjusting for potential confounders. Subgroup analyses according to age, sex, body mass index (BMI), smoking status, and vitamin D levels were performed.

**Results:**

The final population included 331 patients treated with denosumab and 357 controls. COVID-19 incidence was lower in the denosumab group (7.6% vs. 14.6%, p = 0.004). COVID-19 severity was similar in both groups. Multiple logistic regression confirmed an association between denosumab and a reduced occurrence of symptomatic COVID-19 [odds ratio (OR) 0.46, 95% CI 0.21–0.98, p = 0.049]. Subgroup analyses suggested a potential protective effect of denosumab in patients over 75 years (OR 0.12, 95% CI 0.02–0.6, p = 0.011), with a significant interaction between denosumab and age categories (p = 0.047).

**Conclusion:**

Our study confirms that denosumab may be safely continued in COVID-19 patients. RANK/RANKL inhibition seems associated with a reduced incidence of symptomatic COVID-19, particularly among the elderly.

## 1 Introduction

Denosumab is a monoclonal antibody preventing the interaction between receptor activator of nuclear factor kappa-B (RANK) and its ligand (RANKL), ultimately inhibiting osteoclastogenesis (1, 2). RANK and RANKL are also expressed at different levels in the immune system (3) and are especially involved in T-cell activation (4). Hence, denosumab, a RANKL inhibitor, has a potential effect on immune cells that has raised concerns about the safety of this drug. However, it is still unclear whether denosumab increases infection risk. A recent meta-analysis (5) showed a mild increased risk of serious adverse events of infections with denosumab, whereas studies on murine models (6, 7) suggested a protective effect of RANKL inhibition in microbial infections.

Given these limited and divergent findings on the effects of denosumab on the immune system, during the recent coronavirus disease 2019 (COVID-19) pandemic, few authors investigated the hypothesis of a possible impact of denosumab treatment on COVID-19. In an early survey study, women with postmenopausal osteoporosis receiving denosumab were not reported at higher risk for COVID-19 (8). A subsequent Spanish observational study found a negative association between denosumab treatment and COVID-19 incidence (9), and a nationwide registry study reported that denosumab therapy was not associated with worse COVID-19 outcomes (10).

Our study aimed to further characterize these findings, comparing patients in therapy with denosumab with a group of controls, to address the potential association between RANKL inhibition and COVID-19.

## 2 Methods

This study spans a period of 1 year, from 1 January 2020 to 1 January 2021, the time COVID-19 pandemic first and second waves affected Italy, prior to the introduction of severe acute respiratory syndrome coronavirus 2 (SARS-CoV-2) vaccines. Consecutive patients referred to the general endocrinology or the endocrine bone disease outpatient clinic of the Endocrinology Department in Papa Giovanni XXIII Hospital, Bergamo, Italy, were eligible for the study.

The denosumab group included patients with osteoporosis having started denosumab at least 3 months before the enrollment period. The control group included patients referred to the general endocrinology outpatient clinic with other diagnoses or osteoporotic patients who had not yet started any therapy.

Exclusion criteria were i) conditions potentially affecting the immune system (i.e., HIV or history of transplant), ii) treatment for osteoporosis other than denosumab, and iii) treatment with denosumab started less than 3 months before the enrollment period. Finally, patients who did not provide consent for the participation in research activities were excluded from the study.

For each patient, epidemiological, clinical, and drug information was recorded during the scheduled follow-up evaluation for his or her clinical condition. The occurrence and severity of COVID-19 were retrospectively recorded during one or more visits in 2020 and at the following scheduled visit in 2021. If a patient did not attend the in-person visit, data were obtained through a phone call. COVID-19 occurrence was defined as i) a positive swab for SARS-CoV-2 RNA plus associated symptoms and/or ii) COVID-19 diagnosis in a close contact plus specific symptoms such as dyspnea, ageusia, and anosmia. The severity of COVID-19 was determined by the presence of one or more of the following: pneumonia (confirmed by imaging), oxygen therapy, and hospitalization.

We used descriptive statistics to summarize the features of the study population according to denosumab therapy and multivariate logistic regression to assess the relationship between denosumab and COVID-19, adjusting for confounders. Subgroup analyses were performed to assess the relationship between denosumab and COVID-19 according to age (above or below 75 years), sex, body mass index (BMI) (above or below 25 kg/m^2^), smoking status, vitamin D levels (low: below 30 ng/mL; normal: equal to or above 30 ng/mL). We analyzed the available data for each specific analysis, ignoring missing values in other variables. For all tested hypotheses, two-sided p-values ≤0.05 were considered significant. Statistical analysis was performed using STATA Software, release 15.1 (StataCorp LP, College Station, TX, USA).

Sex, diabetes mellitus, smoking status, heart disease, and corticosteroid use were evaluated as dichotomous variables. In particular, heart disease was defined by the presence of heart failure of any cause or history of ischemic heart disease, and corticosteroid use was defined by treatment with ≥5 mg of prednisolone for a duration of ≥3 months. Regarding the smoking status, for the purpose of the regression analysis, former smokers were grouped with nonsmoker.

Age (years), BMI (kg/m^2^), and vitamin D (ng/mL) were recorded and analyzed as continuous variables for the multivariate logistic regression.

## 3 Results

**Figure 1:**
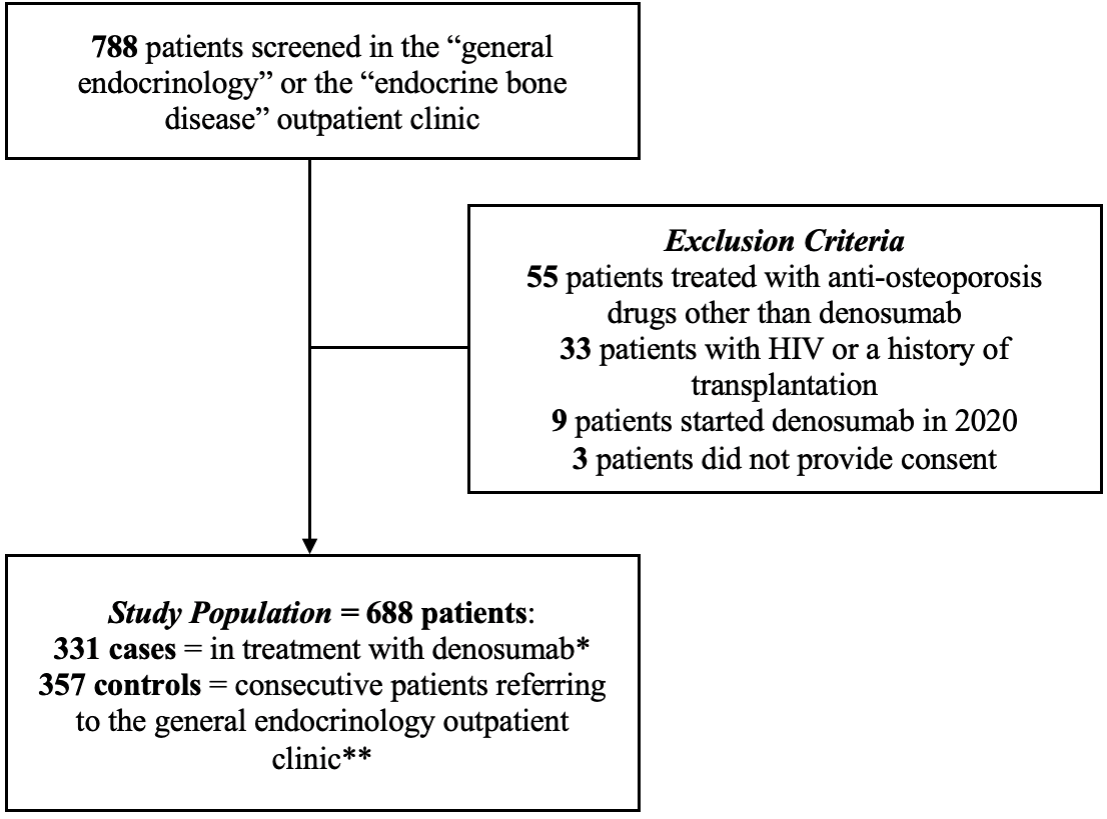
Flow chart of screened, included, and excluded patients. * Started at least 3 months before enrollment period. ** Not currently undergoing osteoporosis treatment.

During the study period, 788 consecutive patients were evaluated; 55 patients treated with anti-osteoporosis drugs other than denosumab, 9 patients who initiated denosumab in 2020, 33 patients with either a history of HIV or a previous transplantation, and 3 patients who did not provide informed consent were excluded. The final population consisted of 688 patients, 331 treated with denosumab and 357 controls. **Figure 1** depicts the flowchart of screened, included, and excluded patients.

### 3.1 Descriptive statistics

**Table 1** summarizes the clinical characteristics of patients and descriptive statistics comparing patients treated with denosumab and controls. Of 688 patients, 82.0% were women: 87.9% in the denosumab group and 76.5% in controls (p < 0.001). Patients in the denosumab group were older than controls (69.4 years, 95% CI 68.2–70.7 vs. 57.7 years, 95% CI 56.2–59.3, p < 0.001); vitamin D levels were higher in the denosumab group as compared to controls (44.2 ng/mL, 95% CI 42.8–45.5 vs. 34.0 ng/mL, 95% CI 31.3–36.7, p < 0.001). There was no difference between the denosumab group and controls regarding BMI (25.9 kg/m^2^, 95% CI 24.4–27.5 vs. 25.7 kg/m^2^, 95% CI 24.9–26.4, p = 0.787), current (16.4% vs. 13.5%) or history (21.5% vs. 19.2%) of smoking (p = 0.368), diabetes mellitus (10.0% vs. 11.5%, p = 0.522), heart disease (7.3% vs. 11.5%, p = 0.058), and current corticosteroid use (7.6% vs. 7.3%, p = 0.893).

**Table 1:** Clinical characteristics of patients.

	Denosumab (N=331)	Controls (N=357)	p*-*value*
**Female sex** N, % (of 688)	291, 87.9%	273, 76.5%	**<0.001**
**Age (years)** Mean, 95% CI (of 688)	69.4 (68.2-70.7)	57.7 (56.2-59.3)	**<0.001**
BMI **(kg/m^2^)** Mean, 95% CI (of 598)	25.9 (24.4-27.5)	25.7 (24.9-26.4)	0.787
Smoke Current History N, % (of 644)	51, 16.4% 67, 21.5%	45, 13.5% 64, 19.2%	0.368
Diabetes mellitus N, % (of 688)	33, 10.0%	41, 11.5%	0.522
Heart disease N, % (of 688)	24, 7.3%	41, 11.5%	0.058
**Vitamin D (ng/mL)** Mean, 95% CI (of 442)	44.2 (42.8-45.5)	34.0 (31.3-36.7)	**<0.001**
Corticosteroids use N, % (of 688)	25, 7.6%	26, 7.3%	0.893
**COVID-19** N, % (of 688) - Pneumonia (confirmed by imaging) - Oxygen therapy - Hospitalization	25, 7.6% 5 of 25, 20% 1 of 25, 4% 1 of 25, 4%	52, 14.6% 13 of 52, 25% 4 of 52, 7.7% 3 of 52, 5.8%	**0.004** 0.627 0.538 0.743

Bold, statistically significant differences (p < 0.05) between patients treated with denosumab and controls. * Based on Student’s t-test or χ² comparisons. BMI, Body Mass Index.

COVID-19 occurrence in 2020 was lower in the denosumab group as compared to controls (7.6% vs. 14.6%, p = 0.004). No significant differences in COVID-19 severity indexes (radiological pneumonia, oxygen therapy, hospitalization) were detected.

### 3.2 Multiple logistic regression

Multiple logistic regression (**Table 2**) confirmed an association between denosumab treatment and a reduced occurrence of symptomatic COVID-19 [odds ratio (OR) 0.46, 95% CI 0.21–0.98, p = 0.049]. Older age was associated with a lower incidence of COVID-19 (OR 0.96, 95% CI 0.93–0.99, p = 0.018), and corticosteroid use was associated with a higher incidence of the disease (OR 3.55, 95% CI 1.24–10.14, p = 0.018). Sex, BMI, smoking, vitamin D levels, diabetes mellitus, and heart disease did not correlate with the COVID-19 incidence.

**Table 2:** Multiple logistic regression to assess factors associated with COVID-19.

COVID-19	Odds Ratio	95% CI	p-value
**Denosumab**	0.46	0.21 - 0.98	**0.049**
**Age *(years)***	0.96	0.93 - 0.99	**0.018**
Sex *(male)*	0.71	0.24 - 2.12	0.543
BMI (*kg/m^2^)*	1.00	0.97 - 1.03	0.998
Smoke	0.81	0.59 - 1.13	0.220
Vitamin D (*ng/mL)*	1.01	0.98 - 1.04	0.378
**Corticosteroids**	3.55	1.24 - 10.14	**0.018**
Diabetes mellitus	0.52	0.11 - 2.39	0.401
Heart disease	1.89	0.55 - 6.53	0.311

Bold, statistically significant differences (p < 0.05). Age, BMI, and vitamin D are assessed as continuous variables, and OR refers to one unit change. BMI, Body Mass Index.

### 3.3 Subgroup analysis

Subgroup analyses (**Table 3**) showed at the univariate logistic regression an association between denosumab treatment and a reduced occurrence of symptomatic COVID-19 in patients aged over 75 years (OR 0.12, 95% CI 0.02–0.6, p = 0.011), with a significant p for interaction for the older age group (0.047). This suggests that the effect of denosumab on COVID-19 may vary significantly across different age groups, with a noteworthy interaction effect observed specifically in the older population. Indeed, denosumab was significantly associated with a reduced occurrence of symptomatic COVID-19 in the following categories: female sex (OR 0.49, 95% CI 0.28–0.85, p = 0.011), overweight/obese BMI category (OR 0.40, 95% CI 0.20–0.77, p = 0.007), and nonsmokers (OR 0.54, 95% CI 0.29–1.00, p = 0.050). However, p for interaction between denosumab and these subgroups was nonsignificant, suggesting no evidence of a distinct impact of denosumab on COVID-19 depending on sex, BMI, or smoking subgroups.

**Table 3:** Subgroup analysis to evaluate the impact of denosumab on symptomatic COVID-19.

COVID-19 - denosumab Subgroups	Odds Ratio	95% CI	p-value*	Interaction p-value**
Age category				
**Age <75**	0.68	0.40 - 1.16	0.161	**0.047**
**Age** ≥75	0.12	0.02 - 0.61	**0.011**	
Sex category				
**Female**	0.49	0.28 - 0.85	**0.011**	0.225
**Male**	0.44	0.12 - 1.65	0.225	
BMI				
**≤25 kg/m^2^**	0.75	0.33 - 1.70	0.495	0.241
**>25 kg/m^2^**	0.40	0.20 - 0.77	**0.007**	
Smoke				
**Nonsmokers**	0.54	0.29 - 1.00	**0.050**	0.975
**Smokers**	0.64	0.13 - 3.03	0.574	
Vitamin D				
**<30 ng/m**L	0.56	0.30 - 1.01	0.057	0.665
**≥30 ng/m**L	0.43	0.15 - 1.18	0.103	

Bold, statistically significant differences (p < 0.05). *p of the univariate logistic regression to assess the relationship between denosumab and COVID-19 in different subgroups. **p for interaction between denosumab and subgroup variables. BMI, Body Mass Index.

## 4 Discussion

Our study suggests that treatment with denosumab may be correlated with a reduced risk of developing symptomatic COVID-19, with no effect on the outcome of the disease. This potential protective effect of denosumab seems to be more prominent in older osteoporotic patients.

### 4.1 Denosumab is associated with a diminished risk of developing COVID-19 symptoms

Few studies so far evaluated the relationship between anti-osteoporotic drugs and COVID-19. Our results confirm those of Blanch-Rubio et al. (9), reporting a negative association between denosumab treatment and COVID-19 incidence in a cohort of 264 patients attending a Rheumatology Service during the COVID-19 pandemic. Our data are also in line with the study of Atmaca et al. (10), showing no effect of denosumab treatment on COVID-19-related outcomes in 195 osteoporotic women over 50 years. It seems therefore that the interaction between denosumab and the RANK/RANKL system may potentially influence the development of COVID-19 symptoms but has no effect on the more severe forms of the disease. However, a possible correlation between denosumab and COVID-19 severity could have been masked by the limited number of patients with severe manifestations of the disease in our population.

### 4.2 Immunomodulatory role of denosumab

The RANK/RANKL system, apart from its role in bone metabolism, acts as a regulator of the immune response (4). RANKL inhibition may potentially interfere with this pathway; hence, concerns of a higher risk of infection in denosumab-treated patients have been raised (5). Conversely, in our study, denosumab seemed to mitigate the risk of developing COVID-19 symptoms. We must however consider that COVID-19 manifestations have been consistently associated with the hyperinflammatory state caused by cytokine overproduction (11). It has been reported that denosumab may decrease the activity of pro-inflammatory cytokines, thus exerting a potential immunomodulatory role (12). Our hypothesis is that denosumab, by inhibiting RANKL, may have hampered the pro-inflammatory state associated with SARS-CoV-2 infection, leading to the decrease in the incidence of symptomatic COVID-19 cases. Further studies are needed to prove this potential protective effect and confirm a causal relationship between denosumab treatment and a reduced occurrence of COVID-19.

### 4.3 Denosumab is associated with a reduced susceptibility to COVID-19 in the elderly

The immunomodulatory hypothesis may also explain the prominent protective role of denosumab in the elderly population. It has been shown that inflammation, senescence, and osteoporosis are in fact strongly linked (13). It is thus possible that elderly osteoporotic patients with a higher basal inflammatory state are more prone to develop manifestations of SARS-CoV-2 infection, as suggested by a recent study highlighting an association between osteoporosis and COVID-19 (14). Denosumab may therefore be able to better modulate the hyperinflammatory state in this population, preventing the complications of an unleashed immune response and ultimately reducing disease manifestations. The diminished susceptibility to COVID-19 among the elderly is also extremely relevant considering the greater risk of in-hospital death in this category of patients when hospitalized for SARS-CoV-2 infection. At the peak of COVID-19 in our hospital, the only independent predictors of mortality were indeed advanced age and severity of the disease (15). Whether these findings on the potential protective effect of denosumab could be extended to other viral infections or to other hyperinflammatory status remains to be clarified both by basic science studies, to better elucidate the molecular mechanisms implied, and by large observational case/control studies, to confirm the outcomes in the real-world setting.

### 4.4 Limitations

Limitations of our study are mainly represented by differences in baseline characteristics of the two study groups. Specifically, the higher percentage of women in the denosumab group may have introduced a bias given the protective role of female sex in SARS-CoV-2 infection. Also, patients were older than controls. These findings were indeed expected, since osteoporosis is much more prevalent in the elderly and women. Finally, patients treated with denosumab had higher vitamin D levels than controls. Given the positive effects of vitamin D on the immune system (16) and its potential protective role in COVID-19 (17), it can be argued that this may have enhanced the beneficial effect of denosumab. Mean vitamin D levels were however within the normal reference range in controls due to our routine to screen and correct vitamin D deficiency also in non-osteoporotic patients. To address these issues, a multivariate analysis adjusted for potential confounders was performed and did not find an interaction between age, sex, or vitamin D levels and COVID-19.

## 5 Conclusions

Our study confirms that denosumab may be safely continued in COVID-19 patients, with a possible reduced susceptibility against symptomatic disease. Considering the potential fracture risk associated with delayed denosumab administration, these findings underlie a significant clinical impact. Older patients may especially benefit from the protective effect of denosumab, suggesting an interaction between RANKL inhibition and the specific mechanisms of inflammation associated with aging.

## Ethical approval

Informed consent was obtained from all individual participants included in the study. Data collection and analysis were approved by an appropriate Institutional Review Board of the local ethical committee. The study was performed in accordance with the Helsinki Declaration of 1964 (and its later amendments) and Good Clinical Practice Guidelines and agreed with national regulations.

## Data availability statement

The raw data supporting the conclusions of this article will be made available by the authors, without undue reservation.

## Ethics statement

The studies involving humans were approved by Review Board of the local ethical committee - Papa Giovanni XXIII. The studies were conducted in accordance with the local legislation and institutional requirements. The participants provided their written informed consent to participate in this study.

## Author contributions

SC: Conceptualization, Data curation, Writing – original draft. SI: Formal Analysis, Methodology, Supervision, Writing – original draft, Writing – review & editing. SP: Conceptualization, Data curation, Writing – original draft. RT: Methodology, Writing – review & editing. AR: Conceptualization, Data curation, Methodology, Supervision, Validation, Writing – original draft, Writing – review & editing.
